# Double Subgenomic Alphaviruses Expressing Multiple Fluorescent Proteins Using a *Rhopalosiphum padi* Virus Internal Ribosome Entry Site Element

**DOI:** 10.1371/journal.pone.0013924

**Published:** 2010-11-10

**Authors:** Michael R. Wiley, Lisa O. Roberts, Zach N. Adelman, Kevin M. Myles

**Affiliations:** 1 Fralin Life Science Institute, Department of Entomology, Virginia Tech, Blacksburg, Virginia, United States of America; 2 Faculty of Health and Medical Sciences, University of Surrey, Guildford, United Kingdom; Institut Pasteur, France

## Abstract

Double subgenomic Sindbis virus (dsSINV) vectors are widely used for the expression of proteins, peptides, and RNA sequences. These recombinant RNA viruses permit high level expression of a heterologous sequence in a wide range of animals, tissues, and cells. However, the alphavirus genome structure and replication strategy is not readily amenable to the expression of more than one heterologous sequence. The *Rhopalosiphum padi* virus (RhPV) genome contains two internal ribosome entry site (IRES) elements that mediate cap-independent translation of the virus nonstructural and structural proteins. Most IRES elements that have been characterized function only in mammalian cells but previous work has shown that the IRES element present in the 5′ untranslated region (UTR) of the RhPV genome functions efficiently in mammalian, insect, and plant systems. To determine if the 5′ RhPV IRES element could be used to express more than one heterologous sequence from a dsSINV vector, RhPV 5′ IRES sequences were placed between genes for two different fluorescent marker proteins in the dsSINV, TE/3′2J/mcs. While mammalian and insect cells infected with recombinant viruses containing the RhPV sequences expressed both fluorescent marker proteins, only single marker proteins were routinely observed in cells infected with dsSINV vectors in which the RhPV IRES had been replaced by a luciferase fragment, an antisense RhPV IRES, or no intergenic sequence. Thus, we report development of a versatile tool for the expression of multiple sequences in diverse cell types.

## Introduction

Alphaviruses (family *Togaviridae*) have a positive strand, non-segmented, RNA genome ∼12 kilobases (kb) in length. The first two-thirds of the genome encode the nonstructural or replicase proteins, while the 3′ one-third encodes the structural proteins. In the infected cell, the 49S genomic RNA serves both as mRNA for the translation of the nonstructural proteins and as a template for synthesis of full-length minus strand RNA copies [1]. The structural proteins are translated from the subgenomic 26S mRNA, which is transcribed from an internal promoter present in the minus strand RNA [1]. This genome structure and replication strategy is amenable to the construction of expression vectors.

Replication and packaging competent alphavirus vectors have been developed by duplicating the subgenomic RNA promoter element in the genome [2], [3]. Heterologous sequences can be expressed as an additional subgenomic RNA transcribed from the duplicated promoter. Double subgenomic alphavirus vectors have several advantages as transient expression systems. These include a tremendously broad host range (e.g. vertebrates and invertebrates), routine construction and manipulation with standard recombinant DNA techniques, and high level expression of proteins, peptides, and RNA sequences [2]. However, expression levels typically diminish with virus passage because of instability in the region of the genome containing the duplicated promoter and heterologous sequence [3], [4]. The utility of alphavirus vectors is also limited by an inability to express more than a single exogenous gene or sequence from the subgenomic promoter. This has previously been addressed by inserting the foot-and-mouth disease virus (FMDV) 2A protein between the N-terminal capsid and PE2 glycoprotein encoded in the 26S mRNA [5]. The alphavirus capsid protein autoproteolytically cleaves itself from the structural polyprotein [6]. The 20 amino acid FMDV 2A sequence mediates self-processing through a proposed ribosomal-skip mechanism [7]. Thus, a 2A fusion protein located at this position in the viral genome can be expressed as a discrete product from the structural polyprotein [5]. Although a protein expressed from the duplicated subgenomic promoter will be in native form, the other protein is always expressed in conjunction with the FMDV 2A peptide sequence [5]. This limits the usefulness of these vectors for some applications, not least of which is the expression of proteins that exhibit reduced bioactivity as fusion products.

An alternative mechanism for achieving the expression of more than one protein from a single mRNA is the insertion of a viral internal ribosome entry site (IRES) element between the two open reading frames (ORFs). An IRES directs a cap-independent mechanism of protein synthesis and therefore efficient expression of both ORFs can be achieved. IRES elements found within the 5′ untranslated regions (UTRs) of picornavirus genomes have been extensively studied for this purpose and are able to direct efficient translation of a downstream ORF within a discistronic mRNA within mammalian cells [8]. As such, much interest has been focused on the use of picornavirus IRES elements in protein expression systems. While these elements have been effectively used in alphavirus expression systems, the mammalian picornavirus IRES elements do not function efficiently in insect cell systems [9], [10], [11]. This limits the usefulness of alphavirus expression systems in dipterans (fruit flies and mosquitoes) and lepidopterans [12], [13], [14]. Here, we have employed an IRES element found within the genome of *Rhopalosiphum padi* virus (RhPV), a virus belonging to the *Dicistroviridae* family. These insect viruses share many characteristics with the *Picornaviridae* but they possess a dicistronic genome, each ORF preceded by an IRES element. However, the function and structure of these IRES elements is very distinct [15]. Unlike the picornavirus IRES elements, the IRES element found within the 5′ UTR of the RhPV genome functions efficiently in insect, mammalian, and plant systems [11], [16], [17]. Further, its utility within a baculovirus protein expression system [18] and a bunyamwera virus replicon system [19] has previously been demonstrated.

Thus, the ability of the RhPV 5′ IRES element to function in insect cells prompted us to assess its ability to initiate translation of a native protein from subgenomic transcripts expressed from the double subgenomic Sindbis virus (dsSINV) vector, TE/3′2J virus [3]. We report high level expression of multiple heterologous sequences from recombinant dsSINV vectors containing the RhPV 5′ IRES element in both insect and mammalian systems, validating the use of this IRES in alphavirus expression vectors.

## Materials and Methods

### Plasmid construction

**Figure 1:**
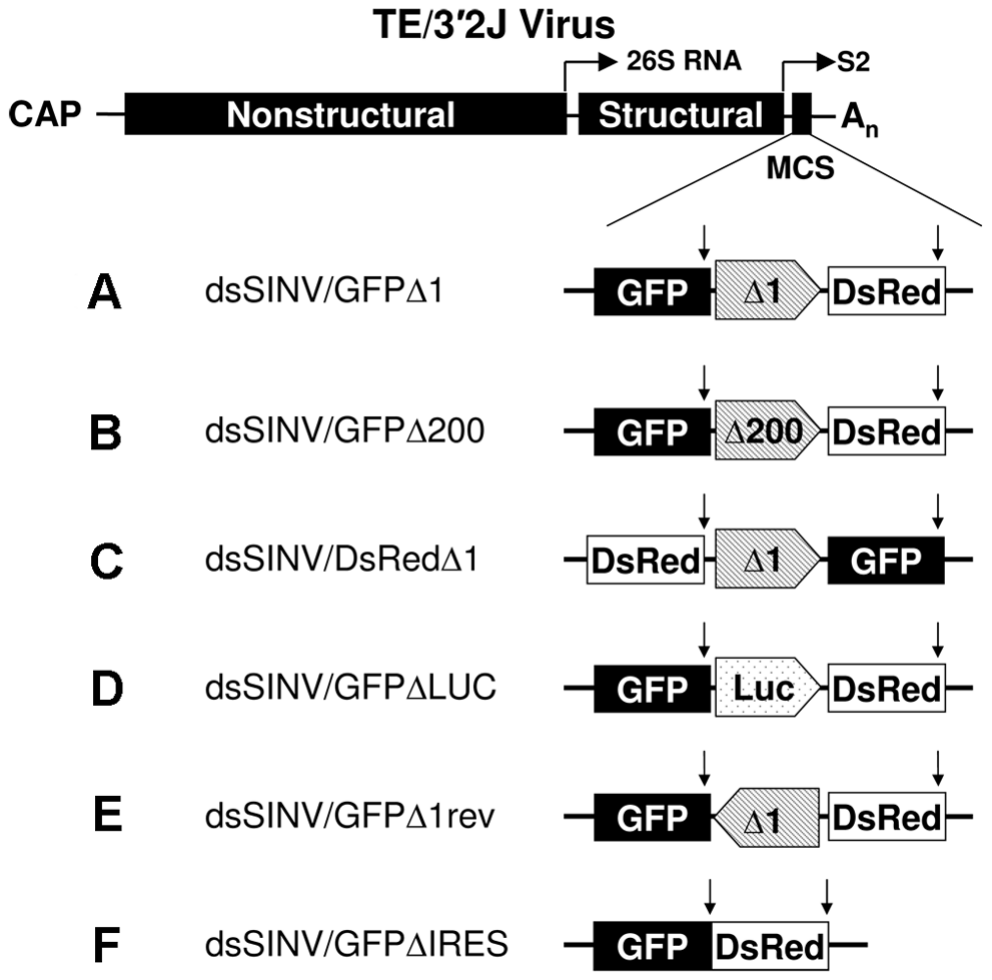
Recombinant viruses generated from pTE/3′2J/mcs. The complete 5′ UTR of the RhPV genome containing the IRES element, (A) Δ1, or a truncated 5′ UTR, (B) Δ200, was inserted between the *Aequorea coerulescens* green fluorescent protein (GFP) or *Discosoma* red fluorescent protein (DsRed) ORFs, downstream from the second subgenomic promoter of TE/3′2J. (C) A third construct reversed the order of GFP and DsRed in relation to the Δ1 sequence. Virus constructs containing a fragment of the (D) firefly luciferase (LUC) gene, (E) the Δ1 sequence in the antisense orientation, or (F) a construct lacking any intergenic sequence between GFP and DsRed served as negative controls. Vertical arrows denote the location of stop codons in reporter gene ORFs.

Virus constructs were generated from a modified pTE/3′2J [3] in which a multiple cloning site (mcs) had been added [20]. The coding sequence for *Aequorea coerulescens* green fluorescent protein (*AcGFP*) or *Discosoma* red fluorescent protein (*DsRed*) was inserted into the *Asc*I and *Pac*I sites of pTE/3′2J/mcs. RhPV 5′ IRES element sequences were amplified from the previously described pGEM-CAT/RhPVΔ1/LUC plasmid [11]. These sequences were subcloned into a pSLfa plasmid [21], previously modified by digestion with *Bam*HI and *Bg*lII, followed by ligation of the compatible ends to remove both restriction sites. RhPV 5′ IRES sequences (in sense or antisense orientation) or other intergenic sequences were amplified and inserted, along with the AcGFP or DsRed ORFs, into the *Xho*I and *Stu*I sites of the modified pSLfa. The RhPV IRES/reporter and control/reporter constructs were then excised from *Pac*I and *Sph*I restriction sites in pSLfa (added into the plasmid by the primer sequences used in the previous step; Table S1). The excised fragments were ligated into the *Pac*I and *Sph*I sites of either pTE/3′2J/GFP or pTE/3′2J/DsRed. This gave rise to the recombinant viruses dsSINV/GFP-Δ1DsRed, dsSINV/GFP-Δ200DsRed and dsSINV/DsRed-Δ1GFP containing the RhPV 5′ IRES element in the sense orientation between the two ORFs, dsSINV/GFP-revΔ1DsRed containing the IRES element in the antisense orientation, and dsSINV/GFP-ΔLUCDsRed and dsSINV/GFP-DsRed containing no IRES element (Fig. 1). All PCR amplifications were performed with Platinum® Pfx polymerase (Invitrogen). A complete list of the primer sequences used in the construction of recombinant viruses is provided in Table S1.

### Cells and viruses

*Aedes albopictus* mosquito (C6/36), baby hamster kidney (BHK- 21), and African green monkey kidney (Vero) cells were obtained from ATCC. Cells were maintained in DMEM supplemented with penicillin, streptomycin, L-glutamine, and 10% fetal bovine serum at 37°C (BHK-21 and Vero cells) or 28°C (C6/36 cells). Recombinant viruses were rescued as described previously [22]. Virus titers were determined in triplicate by plaque assay on Vero cell monolayers.

### Infection of cells and mosquitoes

Cells were grown in 25 cm^2^ tissue culture flasks, washed and infected with virus at a multiplicity of infection (MOI) of 0.05. Virus stocks were diluted with DMEM, placed on cells, and rocked for one hour at RT. After one hour the inoculum was removed, cells were washed three times with PBS, and fresh medium added to each flask. Aliquots (300 µl) of the culture supernatant were taken every 12 hours, and virus titers were determined by plaque assay. Mosquito colonies were reared at 28°C, 70% relative humidity, with a photoperiod of 14 hours light/10 hours dark. One to two day old female white-eyed *Aedes aegypti* (*kh* ^w^) were injected with a suspension of recombinant virus (∼500 pfu/mosquito) and examined with a Leica MZ-16FL stereofluorescence microscope for eye-specific fluorescence at 1, 2, 3, 4 and 7 dpi.

Virus stability was assessed by plaque assay as described previously [4]. In addition, viral RNA was analyzed by Northern blot using standard procedures. Probes were generated with the Megaprime™ DNA Labeling System (Amersham) from a fragment spanning the *Xba*I and *Xho*I sites of pTE/3′2J/mcs.

### Western blots

Mosquito cells were infected with recombinant viruses at an MOI of 1, as described above. Examination of the cells for GFP-specific fluorescence confirmed that infection was near 100%. Cells were counted (1.5×10^6^) and lysed in 750 µl of 2× SDS loading dye (Novagen). Recombinant AcGFP standards (Clontech) and cell lysates were analyzed by 10% SDS-PAGE, and proteins transferred to a 0.45 µm nitrocellulose membrane using a Mini-PROTEAN®3 system (Biorad). Ponceau S (Sigma) staining was used to confirm complete transfer of samples to the membrane, as well as equal loading. Membranes were probed with a mouse anti-AcGFP monoclonal primary antibody (Clontech) as per the manufacturer's instructions, followed by a goat anti-mouse horseradish peroxidase conjugate (Calbiochem) as per the manufacturer's instructions. Fluorescence was detected with ECL Plus (Amersham) on a Storm 840 phosphorimager (GE Healthcare), and quantified with ImageQuant software (GE Healthcare). For protein quantification, samples of unknown GFP concentration were loaded in triplicate along with the GFP standards of known concentration. The amount of GFP in the unknown samples was then determined from standard curves generated from the known quantities of GFP.

## Results

### Expression of multiple heterologous proteins from recombinant dsSINV vectors

The complete 579 nt 5′ UTR of the RhPV genome containing the IRES element, RhPVΔ1 [11], was inserted between the GFP and DsRed coding sequences, downstream from the second subgenomic promoter of the dsSINV, TE/3′2J (Fig. 1A). Because it has been postulated that the stability of heterologous sequences in double subgenomic alphavirus vectors is inversely related to size [2], [3], a second dsSINV construct was generated that contained a fragment of the RhPV 5′ UTR lacking the 5′ 200 nt (RhPVΔ200, Fig. 1B). The RhPVΔ200 fragment has previously been shown to function efficiently as an IRES element [16]. A third construct reversed the order of GFP and DsRed in relation to the RhPVΔ1 sequence (Fig. 1C). Virus constructs containing a fragment of the firefly luciferase (LUC) gene (Fig. 1D), the RhPVΔ1 sequence in the antisense orientation (Fig. 1E), or a construct lacking any intergenic sequence between GFP and DsRed (Fig. 1F) served as negative controls.

**Figure 2:**
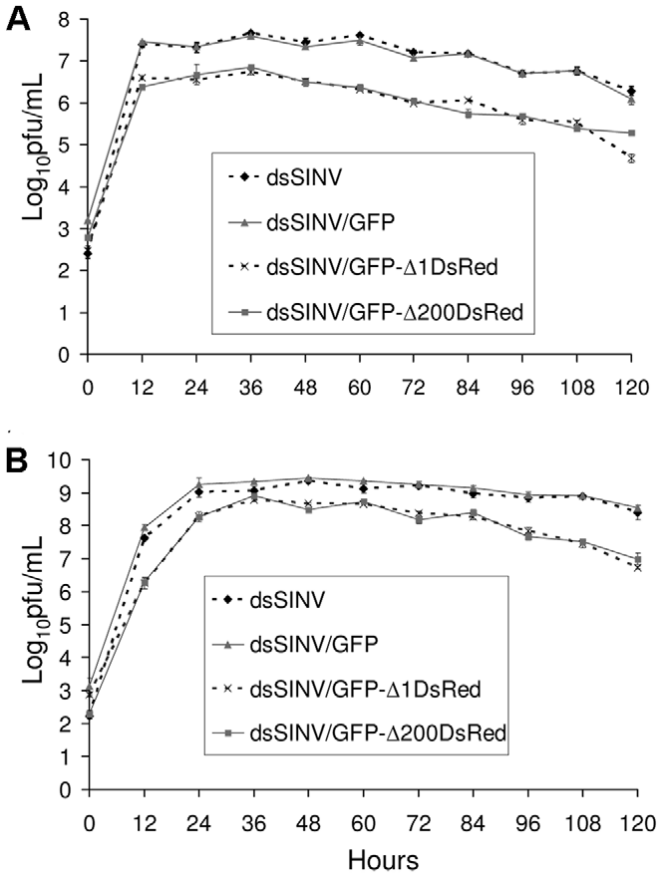
Growth of recombinant dsSINV constructs in BHK-21 (A) or C6/36 (B) cells. Infections were done in triplicate at an MOI of 0.05. Culture supernatant was harvested every 12 hours and virus titers determined by plaque assay. Errors bars indicate one standard deviation among three replicates at each time point.

Initial characterization of recombinant viruses consisted of growth analysis. The growth of recombinant virus containing only GFP (total insert size of 745 nt) was comparable to that of dsSINV containing no insert, in both mammalian (BHK-21) and mosquito (C6/36) cells (Fig. 2A and B). The addition of DsRed and either the full-length or truncated RhPV 5′ UTR to the GFP sequence already present (total insert size of 2029 nt for RhPVΔ1 or 1829 nt for RhPVΔ200), decreased virus production by approximately ten-fold in both cell types (Fig. 2A and B). This may have been due to reductions in packaging efficiency, as the total viral genome size in these constructs may be approaching the upper limit of the virion's packaging capacity [2], [3]. Regardless of the exact mechanism for the reduced number of plaque forming units (pfu) produced in each cell type, viruses containing RhPV sequences still replicated to relatively high levels in both cell types. These viruses reached similar peak titers of ∼6.7 log_10_ pfu/ml in BHK-21 cells, and ∼8.7 log_10_ pfu/ml in C6/36 cells (Fig. 2A and B).

**Figure 3:**
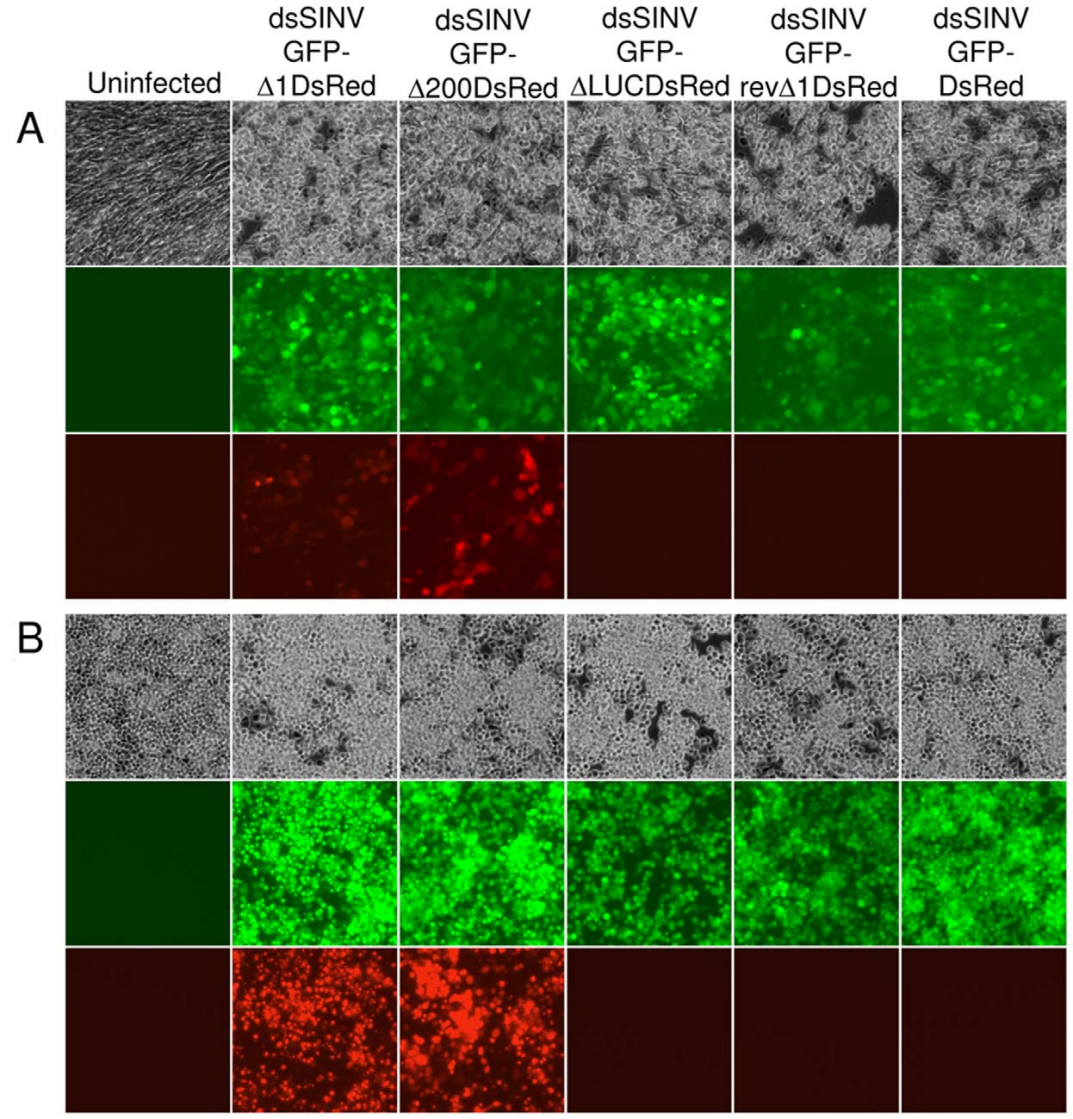
IRES-directed expression of DsRed in cells. (A) BHK-21 or (B) C6/36 cell monolayers were infected in triplicate with recombinant dsSINV constructs. Pictures were taken at 10× magnification using a Zeiss Axiovert epi-fluorescence microscope. White light pictures show monolayer confluency at (A) 3 and (B) 4 dpi (upper panels). GFP-specific fluorescence indicates cap-dependent translation of the first ORF (middle panels). DsRed-specific fluorescence indicates 5′-end-independent translation directed by the RhPV 5′ IRES element (lower panels).

**Figure 4:**
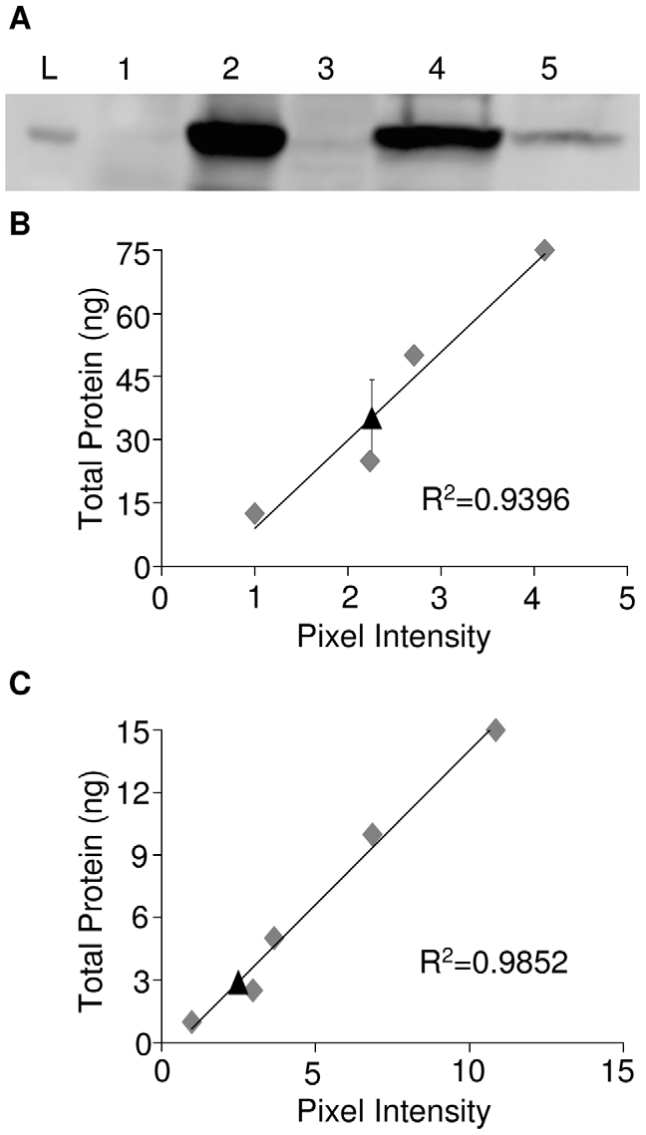
Cap- and IRES- dependent GFP expression levels in mosquito cells. (A) Detection of GFP by Western blot in uninfected C6/36 cells (lane 1), C6/36 cells infected with dsSINV/GFP (lane 2), dsSINV/DsRed (lane 3), dsSINV/GFP-Δ1DsRed (lane 4) or dsSINV/DsRed-Δ1GFP (lane 5) at 3 dpi. A 25 kDa ladder is shown (lane L). The total amount of GFP present in 30 µl of cell lysate was determined from standard curves generated from known concentrations (grey diamonds) of recombinant GFP (Clontech). (B) C6/36 cells infected with dsSINV/GFP-**Δ**1DsRed. Black triangle indicates the amount of GFP produced by cap-dependent translation (35.15 ng). (C) C6/36 cells infected with dsSINV/DsRed-**Δ**1GFP. Black triangle indicates the amount of GFP produced by IRES-dependent translation (2.88 ng). Errors bars indicate one standard deviation among three replicates.

To evaluate the ability of the RhPV 5′ IRES to direct cap-independent translation in the context of an alphavirus subgenomic mRNA transcript, cells infected with recombinant dsSINV constructs were analyzed for the expression of fluorescent reporter proteins. Cap-dependent translation from dsSINV subgenomic transcripts was monitored by GFP expression, while IRES-dependent initiation was monitored by the expression of DsRed. As expected, all of the recombinant dsSINV vectors expressed GFP in both BHK-21 (Fig. 3A) and C6/36 (Fig. 3B) cells. However, efficient expression of DsRed was only observed in cells infected with viruses containing the RhPVΔ1 or RhPVΔ200 sequences in the sense orientation (Fig. 3A and B). RhPVΔ1-directed expression of DsRed over multiple time points is shown in Figure S1. Little or no DsRed expression was observed in cells infected with viruses lacking any intergenic sequence between the two reporter proteins (Fig. 3A and B). Similarly, cells infected with viruses containing the antisense RhPVΔ1 sequence, or LUC fragment in the intergenic region, also exhibited almost no DsRed-specific fluorescence (Fig. 3A and B). Internal initiation of translation directed by the RhPVΔ1 sequence was determined to be approximately 12 times less efficient than cap-dependent initiation of translation in dsSINV-infected C6/36 cells at three days post-infection (Fig. 4). By three days post-infection, cells infected with dsSINV/GFP-**Δ**1DsRed (cap-dependent translation) had accumulated ∼1.31×10^7^ molecules of GFP per cell, while those infected with dsSINV/DsRed-**Δ**1GFP (IRES-dependent translation) accumulated ∼1.07×10^6^ molecules per cell (Fig 4B and C).

**Figure 5:**
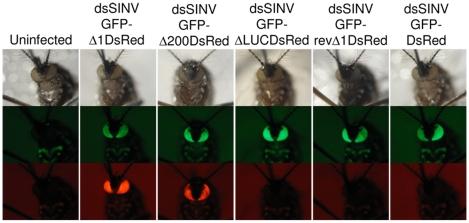
IRES-directed expression of DsRed in mosquitoes. *Aedes aegypti* were injected with recombinant dsSINV constructs and pictures were taken 3 dpi. White light pictures of mosquito eyes (upper panels). GFP-specific fluorescence indicates cap-dependent translation (middle panels). DsRed-specific fluorescence indicates 5′-end-independent translation directed by the RhPV 5′ IRES element (lower panels).

Several studies have successfully employed alphavirus vectors to express heterologous proteins or silence genes in a range of medically important mosquito vector species [4], [13], [23], [24], [25]. To determine the utility of dsSINV vectors containing RhPV IRES elements in a mosquito, adult white-eyed *Aedes aegypti* were injected with each recombinant virus. As expected, all of the recombinant dsSINV vectors expressed GFP in the eyes of infected mosquitoes (Fig. 5). However, expression of DsRed was only observed in the eyes of mosquitoes infected with viruses containing the RhPVΔ1 or RhPVΔ200 sequences in the sense orientation (Fig. 5 and Table S2). Thus, we conclude that the RhPV 5′ IRES element can efficiently initiate 5′-end-independent translation of dsSINV subgenomic mRNA transcripts in both mammalian and insect host systems.

### Stability of recombinant dsSINV vectors containing the RhPV 5′ IRES sequences

Repeated passage of double subgenomic alphavirus vectors containing heterologous sequences generally results in the appearance of deletion mutants no longer expressing any functional insert [4]. However, recombinant dsSINV vectors containing heterologous sequences >2 kb tend to be less stable than those with smaller inserts [2], [3]. Deletion variants appear to arise more readily when a larger insert is present, and can represent a substantial portion of the total virus recovered even from an initial transfection [3]. Because this fraction increases with passage [4], deletion variants generally become a larger portion of the total virus population more rapidly when viruses contain larger inserts.

**Figure 6:**
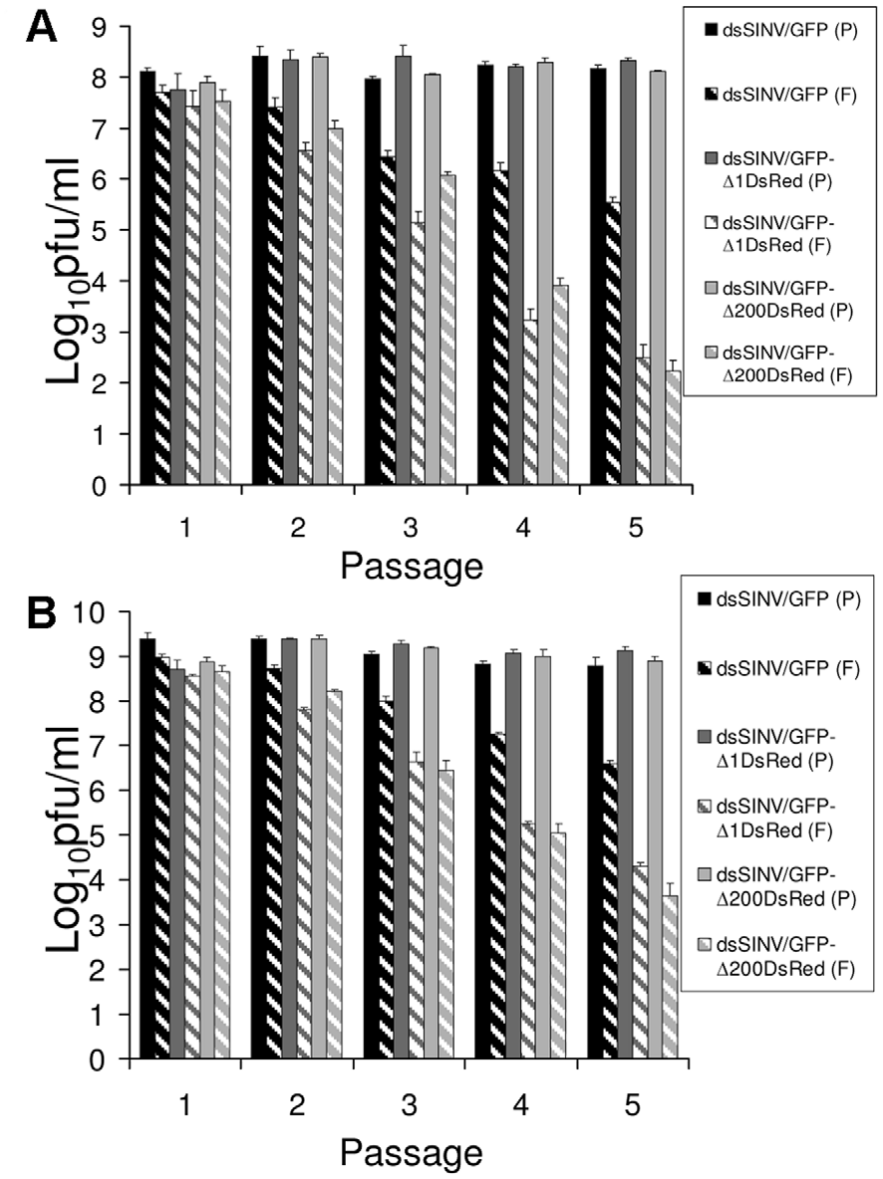
Analysis of recombinant dsSINV stability by plaque assay. Total virus titers determined by (P) plaque assay and (F) “GFP-expressing virus” titers determined by the number of plaques which were GFP positive following passage in (A) mammalian, or (B) mosquito cultured cells. Errors bars indicate one standard deviation among three replicates.

To assess the stability of dsSINV constructs containing RhPV IRES elements, recombinant viruses expressing GFP (Fig. 1A and B), with or without DsRed and associated IRES sequences, were serially passaged in mammalian and insect cells. Plaque assays were used to determine total virus titers and “GFP-expressing virus” titers after each passage. While total virus titers remained relatively constant after each passage (ranging between 7.8–8.4 pfu/ml in BHK-21 cells and 8.7–9.4 pfu/ml in C6/36 cells), the “GFP-expressing virus” titers of all recombinant vectors declined with passage (Fig. 6). Nevertheless, the percentage of total virus that expressed GFP did not differ significantly between viruses, with or without DsRed and the associated IRES element, after one passage in either cell type (p-values ≥ 0.14; One-Way ANOVA). However, comparison at subsequent passages revealed increasingly significant differences (p-values ≤ 0.02), indicating the presence of a greater number of deletion variants in the viruses containing larger inserts. These observations do not appear to be directly related to the IRES element itself, as deletion variants appear to arise at an equivalent rate following passage of virus with a similar total insert size, but containing a fragment of LUC in place of an RhPV sequence (data not shown). These results confirm previous observations indicating double subgenomic alphaviruses containing larger inserts are less stable than viruses containing smaller inserts [2], [3], [4].

**Figure 7:**
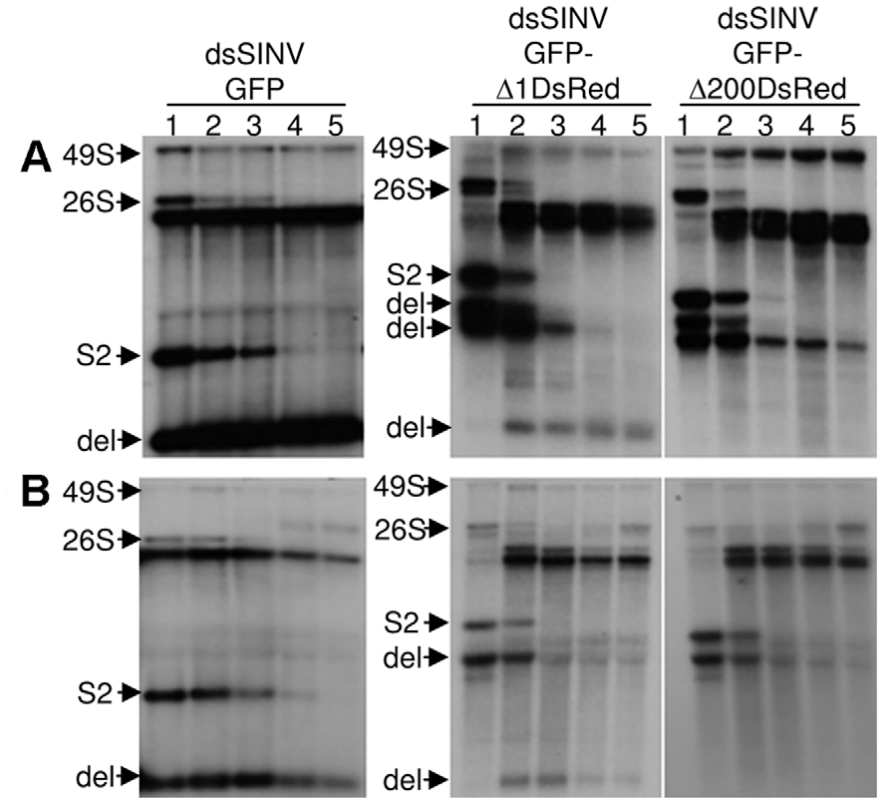
Analysis of recombinant dsSINV stability by Northern blot. Detection of dsSINV RNA following passage (1–5) in (A) mammalian cells, or (B) mosquito cells. Full genomic length RNA (49S), subgenomic mRNA (26S), full-length second subgenomic RNA (S2), and deletion variants (del) are shown. Viral RNA was detected with a ^32^P-label probe generated from a fragment spanning the *Xba*I and XhoI sites of pTE/3′2J/mcs.

Following each passage, Northern blot analysis confirmed that viruses no longer expressing fluorescent marker proteins were in fact deletion variants. Consistent with the plaque assay results, deletion variants were more readily detected in viruses harboring larger inserts (those containing DsRed and associated RhPVΔ1 or RhPVΔ200 sequences), regardless of cell type (Fig. 7A and B). After the third passage of dsSINV vectors containing DsRed and RhPV sequences, full length subgenomic mRNA (containing a complete dicistronic insert) could not be detected (Fig. 7A and B). In comparison, dsSINV containing only the GFP sequence required four passages before full-length subgenomic mRNA sequences were no longer detected (Fig. 7A and B). Nevertheless, our results suggest that the stability of recombinant dsSINV vectors containing RhPV 5′ IRES sequences is sufficient for most applications of such expression systems.

## Discussion

We have demonstrated expression of two different fluorescent proteins, both in native form, from recombinant dsSINV vectors containing RhPV 5′ IRES sequences. Expression of more than two proteins is theoretically possible from the use of multiple IRES elements, but such an approach may be better suited for replication-competent, packaging defective, replicon vectors with their greater capacity (at least 5 kb) for insertion of heterologous sequences [2]. We estimate 5′-end-independent expression directed by the RhPV IRES element to be in the order of 10^6^ polypeptides per dsSINV-infected cell. Expression vectors containing RhPV IRES elements also appear to have sufficient stability for most applications not requiring extensive passaging of the virus (Table S2 and Fig. S1). Expression of heterologous sequences from a subgenomic promoter positioned upstream of the structural protein genes may further improve the stability of such constructs, facilitating a potentially wider range of applications, albeit at the risk of lower expression levels [3]. Expression of proteins, peptides, and RNAs smaller in sequence than the GFP (720 nt) and DsRed (681 nt) ORFs used in this study may also yield increases in stability.

As previously reported, there was little discernable difference in the IRES activity of the full-length IRES (RhPVΔ1) and the IRES with the 5′ 200 nt deleted (RhPVΔ200) [16]. Although the stability of viruses containing these sequences were also similar, in each case the total size of the inserted heterologous sequences remained near the 2 kb limit previously reported to be optimal for dsSINV vectors [2], [3]. Therefore, the extra coding capacity accommodated by the shorter RhPVΔ200 sequence (379 nt versus the 579 nt full-length IRES) may prove to be more beneficial when the expression of larger sequences (>2 kb) is required. It has also been shown that a fragment of the RhPV 5′ UTR corresponding to the 3′ 153 nt functions with approximately 50% of the activity of the full-length IRES [16]. This fragment may prove useful in reducing the insert size further in the context of a dsSINV vector.

Replication and packaging competent vectors developed from alphavirus genomes have proven to be useful in applications ranging from studies of basic virology to vaccine development [2], [26], [27], [28], [29]. However, these virus vectors have two main disadvantages. The first is an inability to express more than a single heterologous sequence in an infected cell. The second is inherent instability in the region of the viral genome containing the duplicated viral promoter and extrinsic sequence [2]. The first problem had previously been addressed by expressing a second heterologous protein as a fusion product with the FMDV 2A protease from modified virus structural proteins [5]. Incorporating an RhPV IRES element into alphavirus vectors offers an alternative solution that does not require the expression of fusion proteins or modification of the virus structural proteins.

Interestingly, the results reported here may also prove to be beneficial in addressing the second problem commonly associated with replication and packaging competent alphavirus vectors, instability. The stability of a double subgenomic rubella virus (family: *Togaviridae*; genus: *Rubivirus*) was greatly improved by replacing one of the two subgenomic promoters in the expression vector with an IRES from encephalomyocarditis virus [30]. Presumably, the increased stability resulted from the elimination of homologous recombination occurring between the identical subgenomic promoter sequences present in the original construct [30]. Although deletion variants continued to arise by other means, they arose at a much lower rate in comparison to the double promoter viruses [30]. The main disadvantage in the application of a similar strategy to alphavirus expression vectors has been the limited host range of most IRES elements previously characterized. However, replacing one of the subgenomic promoters with an RhPV 5′ IRES element may increase stability, while still preserving a primary advantage of double subgenomic alphavirus expression systems, their broad tropism.

## Supporting Information

Figure S1 IRES-directed expression of DsRed in cells over time. A C6/36 cell monolayer was infected at an MOI of 1 with dsSINV/GFP-Δ 1DsRed. Pictures were taken at 20× magnification using a Zeiss Axiovert epi-fluorescence microscope. White light pictures show monolayer confluency at each time point (upper panels). GFP-specific fluorescence indicates cap-dependent translation (middle panels). DsRed-specific fluorescence indicates IRES-directed translation (lower panels). The days post-infection are indicated above each column.
(1.32 MB DOC)

Table S1 Primer sequences used in the construction of recombinant viruses.
(0.04 MB DOC)

Table S2 Expression of GFP and DsRed in *Aedes aegypti* infected with recombinant Sindbis viruses.
(0.04 MB DOC)
