# Supplementary material for: Double Subgenomic Alphaviruses Expressing Multiple Fluorescent Proteins Using a Rhopalosiphum padi Virus Internal Ribosome Entry Site Element
Source: PLoS One. 2010 Nov 10;5(11):e13924. doi: 10.1371/journal.pone.0013924 (PMC2978087; doi:10.1371/journal.pone.0013924)
Supplement: Figure S1 — IRES-directed expression of DsRed in cells over time. A C6/36 cell monolayer was infected at an MOI of 1 with dsSINV/GFP-Δ 1DsRed. Pictures were taken at 20× magnification using a Zeiss Axiovert epi-fluorescence microscope. White light pictures show monolayer confluency at each time point (upper panels). GFP-specific fluorescence indicates cap-dependent translation (middle panels). DsRed-specific fluorescence indicates IRES-directed translation (lower panels). The days post-infection are indicated above each column. (1.32 MB DOC) [file pone.0013924.s001.doc]

**
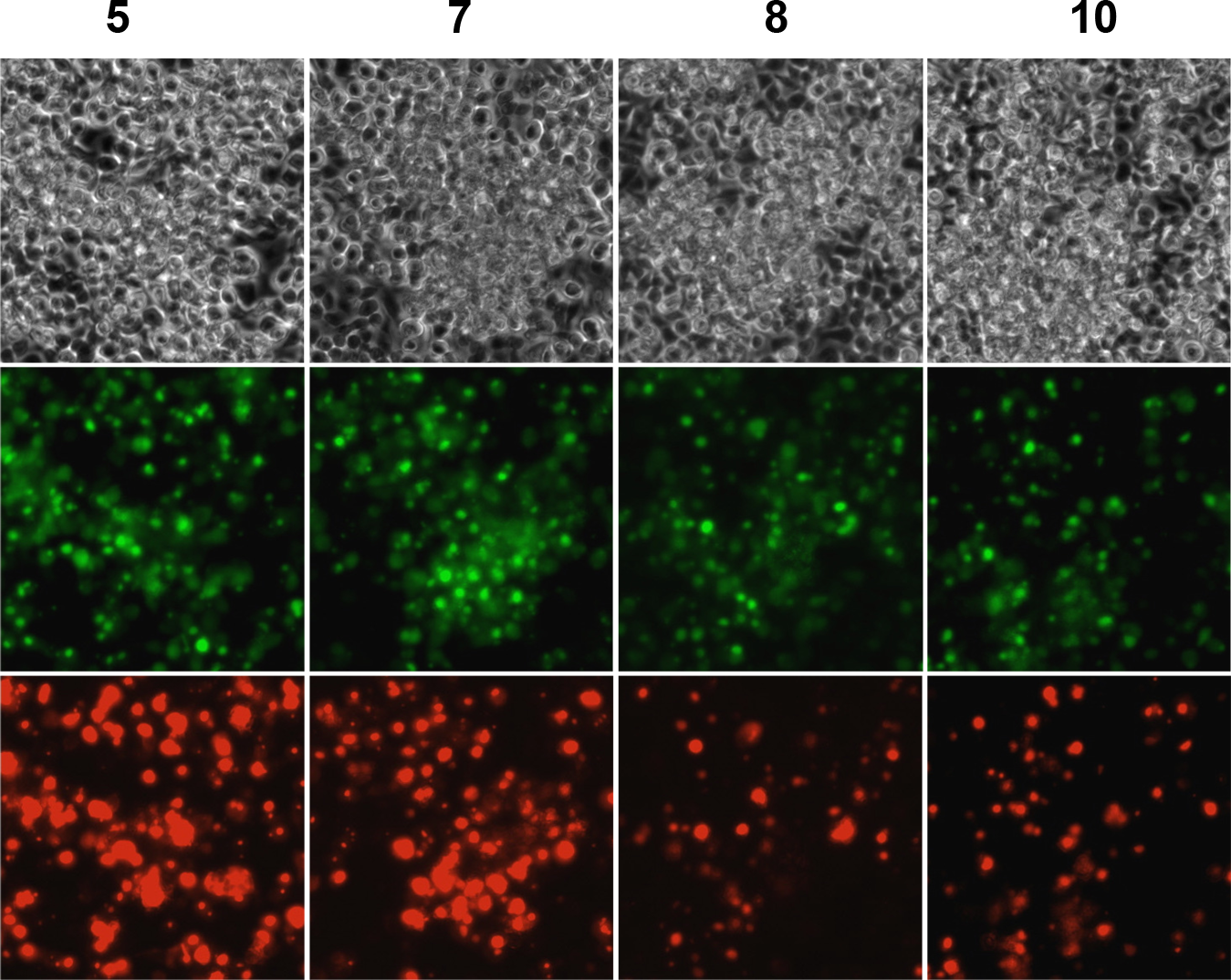
**

**Figure S1. IRES-directed expression of DsRed in cells over time.** A C6/36 cell monolayer was infected at an MOI of 1 with dsSINV/GFP-**∆**1DsRed. Pictures were taken at 20x magnification using a Zeiss Axiovert epi-fluorescence microscope. White light pictures show monolayer confluency at each time point (upper panels). GFP-specific fluorescence indicates cap-dependent translation (middle panels). DsRed-specific fluorescence indicates IRES-directed translation (lower panels). The days post-infection are indicated above each column.
