# Supplementary material for: Double Subgenomic Alphaviruses Expressing Multiple Fluorescent Proteins Using a Rhopalosiphum padi Virus Internal Ribosome Entry Site Element
Source: PLoS One. 2010 Nov 10;5(11):e13924. doi: 10.1371/journal.pone.0013924 (PMC2978087; doi:10.1371/journal.pone.0013924)
Supplement: Table S1 — Primer sequences used in the construction of recombinant viruses. (0.04 MB DOC) [file pone.0013924.s002.doc]

| Supplemental Table S1. Primer sequences used in the construction of recombinant viruses | | | | | | | | | | | |  |
| --- | --- | --- | --- | --- | --- | --- | --- | --- | --- | --- | --- | --- |
|  |  | Forward primers (5'-3') | | | | | | Reverse primers (5'-3') | | | |  |
| RhPV∆1/LUC | | aaactcgagttaattaaGATAAAAGAACCTATAATCCCTTCGCACACC | | | | | | aaaaggcctCGGAGGATTACAATAGCTAAGAAT | | | |  |
| RhPV5’∆200/LUC | | aaactcgagttaattaaAGGACCTTTAAGTCCCTACTATACTA | | | | | | aaaaggcctCGGAGGATTACAATAGCTAAGAAT | | | |  |
| IRES/GFP |  | aaaggatccACCATGGTGAGCAAGGG | | | |  |  | tttgcatgcCACTTGTACAGCTCATCCATGCCGTGGGT | | | |  |
| IRES/DsRed |  | aaaggatccAAATGGTGCGCTCCTCCAAGAAC | | | | |  | tttgcatgcCTACAGGAACAGGTGGTGGCG | | | |  |
| ∆LUC |  | tttctcgagttaattaaCAATTGTTTTGTCACGATCAAAGGAC | | | | | | aaaggatccAAATGGAAGACGCCAAAAACATA | | | |  |
| RhPV∆1 reverse | | ctcgagttaattaaTATAAATAGATAAAGCTAATG | | | | |  | aaaggatccACCTATAATCCCTTCGCACACC | | | |  |
| GFP-IRES |  | aaaggcgcgccACCATGGTGAGCAAGGG | | | | |  | tttttaattaaCACTTGTACAGCTCATCCATGCCGTGGGT | | | | |
| DsRed-IRES |  | aaaggcgcgccAAATGGTGCGCTCCTCCAAGAAC | | | | |  | tttttaattaaCTACAGGAACAGGTGGTGGCG | | | |  |
| Notes. Lowercase letters indicate added endonuclease sites | | | | | | |  |  |  |  |  |  |
|  |  |  |  |  |  | |  |  |  |  |  |  |
