# Supplementary material for: Double Subgenomic Alphaviruses Expressing Multiple Fluorescent Proteins Using a Rhopalosiphum padi Virus Internal Ribosome Entry Site Element
Source: PLoS One. 2010 Nov 10;5(11):e13924. doi: 10.1371/journal.pone.0013924 (PMC2978087; doi:10.1371/journal.pone.0013924)
Supplement: Table S2 — Expression of GFP and DsRed in Aedes aegypti infected with recombinant Sindbis viruses. (0.04 MB DOC) [file pone.0013924.s003.doc]

|  |  |  |  |  |  |  |  |  |  |  |
| --- | --- | --- | --- | --- | --- | --- | --- | --- | --- | --- |
| Supplemental Table S2. Expression of GFP and DsRed in *Aedes aegypti* infected with recombinant Sindbis viruses | | | | | | | | | | |
|  | Number of mosquitoes expressing GFP or DsRed | | | | | | | | | |
| Virus | Day 1 | | Day 2 | | Day 3 | | Day 4 | | Day 7 | |
|  | GFP | DsRed | GFP | DsRed | GFP | DsRed | GFP | DsRed | GFP | DsRed |
| dsSINV/GFP-∆1DsRed | 9/12 | 0/12 | 17/17 | 14/17 | 21/21 | 20/21 | 19/19 | 18/19 | 18/18 | 17/18 |
| dsSINV/GFP-∆200DsRed | 5/10 | 0/10 | 16/16 | 11/16 | 20/20 | 19/20 | 18/18 | 16/18 | 18/18 | 13/18 |
| dsSINV/GFP-∆LUCDsRed | 17/19 | 0/19 | 17/17 | 0/17 | 19/19 | 0/19 | 20/20 | 0/20 | 18/18 | 0/18 |
| dsSINV/GFP-rev∆1DsRed | 13/17 | 0/17 | 18/18 | 0/18 | 16/16 | 0/16 | 19/19 | 0/19 | 19/21 | 1/21 |
| dsSINV/GFP-DsRed | 6/12 | 0/12 | 21/21 | 0/21 | 19/19 | 0/19 | 16/16 | 0/16 | 19/19 | 0/19 |
|  |  |  |  |  |  |  |  |  |  |  |
